# Editorial: The safety, efficacy, and effectiveness of allergen-specific oral immunotherapy

**DOI:** 10.3389/falgy.2024.1448220

**Published:** 2024-08-23

**Authors:** Alexandra Lee, Rosemarie DeKruyff, Luisa Ricciardi

**Affiliations:** ^1^ Sean N. Parker Center for Allergy and Asthma Research at Stanford University, Stanford University, Stanford, CA, United States; ^2^ Operative Unit of Allergy and Clinical Immunology, Department of Clinical and Experimental Medicine, University of Messina, Messina, Italy

**Keywords:** allergy, OIT, sublingualimmunotherapy, food allergy, safety, efficacy

**Editorial on the Research Topic**
The safety, efficacy and effectiveness of allergen-specific oral immunotherapy

Oral immunotherapy (OIT), in which patients with allergy ingest small and increasing amounts of the protein that triggers their allergy under careful medical supervision, has roots that can be traced back to the early 20th century. The underlying mechanism behind OIT relies on the treatment's ability to decrease the sensitivity of dendritic cells. Dendritic cells can produce immunoglobulin E (IgE) antibodies, hence their downregulation is helpful in attenuating immune responses to allergens. OIT is especially useful in treating individuals with severe allergies, as many live in fear of accidental allergen exposure that may result in life-threatening reactions. When applied over an extended period of time, OIT can result in allergen desensitization (i.e., training one's immune system to tolerate a given allergen). Hence, while it is not a curative therapy, OIT has the potential to raise the threshold of an allergenic protein that is needed to trigger a reaction and to protect individuals against accidental exposure.

Currently, there is no OIT treatment approved for multi-food allergens or for allergens other than peanut in the European Union or United States. OIT remains available through clinical research settings, however, where the treatment's frontiers have expanded to include multi-allergen OIT and biologics (i.e., drugs derived from living cells). In order to increase access to OIT and its success in achieving allergen desensitization, more must be known regarding dosing strategies, safety, and mechanisms of immune response in food allergy and allergic diseases. In this Research Topic, the safety, efficacy, and effectiveness of allergen-specific OIT are addressed.

The manufacturing and testing process of allergen-specific OIT products lays a crucial foundation for safety and efficacy. Since there can be a wide range of variability in allergen potency, rigor is necessary to ensure reliable dose consistency and stability. This is particularly important during OIT initial dose escalation and up-dosing (Leonard et al.). In addition, Bukhari et al. assessed the accuracy of SPTs with milk extract, diluted, and undiluted milk to detect desensitization in children with milk allergy undergoing OIT. They found that the mean decrease in wheal size was significant in the active arm but not in the control arm, indicating that milk extract SPTs can effectively monitor sensitization in a desensitization program (Bukhari et al.).

Alternatives to OIT, such as sublingual allergen immunotherapy (SLIT) and epicutaneous immunotherapy (EPIT) were studied. Thétis-Soulié et al. investigated the real-life practice of dose adjustments in SLIT for respiratory allergies. They found that the majority of patients had their doses adjusted, primarily due to adverse events, treatment effectiveness, and allergen sensitivity. Nevertheless, their results demonstrated significant reductions in disease severity for both allergic rhinitis and asthma, regardless of dose adjustments, highlighting the effective nature of precision dosing (Thétis-Soulié et al.). In a study looking at the transition between EPIT and OIT in pediatric patients, it was found that peanut-allergic children and their caregivers reported less anxiety regarding OIT when they had previous exposure through EPIT. Most children in the study who made the transition had mild to no symptoms initially and were also able to maintain or increase their daily OIT dosage over time (Wong et al.).

Subcutaneous immunotherapy (SCIT) was compared to OIT for house dust mite-induced allergic rhinitis. It was shown that OIT was more effective than SCIT in improving symptoms, and local and systemic adverse reactions were observed in the SCIT group though none were reported in the OIT group (Zhang et al.). These findings suggest OIT is a safe and effective form of immunotherapy for allergic rhinitis. A sublingual allergen oral immunotherapy tablet was also shown to be a safe and well-tolerated AR treatment in a cohort of adults in Dutch clinical practice. Adverse effects occurred often but were mostly mild and decreasing during the first year (Tempels-Pavlica et al.).

OIT's efficacy has also been demonstrated in genetically diverse mice models. Seeking to discern whether airway sensitization and oral tolerance to peanuts could be induced in mice, Immormino et al. found that mice could only successfully induce peanut-specific IgE and IgG1 following airway exposure to peanuts without oral peanut feeding (Immormino et al.). There is currently ongoing research to explore the potential efficacy of abatacept, a cytotoxic T lymphocyte-associated antigen-4 (CTLA-4) immunoglobulin fusion protein, in promoting immune tolerance to food allergens during OIT (Braun et al.).

